# Prophylactic Effect of Prefrontal Alternating Current Stimulation on Postoperative Sleep Disturbance in Patients Undergoing Gynecological Laparoscope: A Randomized, Double‐Blind, Sham‐Controlled Trial

**DOI:** 10.1111/cns.70529

**Published:** 2025-08-04

**Authors:** Ni Du, Jin‐Jin Yang, Xin‐Yu Li, Zhi‐Hao Li, Kenji Hashimoto, Lei Lei, Jian‐Jun Yang

**Affiliations:** ^1^ Department of Anesthesiology, Pain and Perioperative Medicine The First Affiliated Hospital of Zhengzhou University Zhengzhou China; ^2^ Henan Province International Joint Laboratory of Pain, Cognition, and Emotion Zhengzhou China; ^3^ Chiba University Center for Forensic Mental Health Chiba Japan

**Keywords:** gynecological laparoscopy, postoperative anxiety, postoperative pain, postoperative sleep disturbance, transcranial alternating current stimulation

## Abstract

**Aims:**

Postoperative sleep disturbance (PSD) is a common complication following surgical procedures. We aimed to evaluate the effect of prefrontal transcranial alternating current stimulation (tACS) in preventing PSD among patients undergoing gynecological laparoscopic surgery.

**Methods:**

A total of 176 eligible patients, aged 18–65 years, with ASA Class I to III, and scheduled for gynecological laparoscopic surgery, were randomly allocated to receive either a single 20‐min session of prefrontal tACS (2 mA, 7 Hz) or sham stimulation immediately after extubation. The primary outcome was the occurrence of PSD on postoperative day (POD) 1.

**Results:**

The intention‐to‐treat analysis showed a statistically significant reduction in PSD incidence on POD 1 in the active tACS group (23.9%) compared to the sham group (43.2%), with an odds ratio of 0.41 (95% CI, 0.22–0.79; *p* = 0.007). Additionally, patients in the active tACS group reported significantly lower anxiety scores on POD 1 (*p* < 0.001), while depression scores were comparable between the groups. The active tACS group also reported significantly lower pain scores, both on PODs 1 (movement: *p* = 0.002; rest: *p* < 0.001) and 3 (movement: *p* = 0.028; rest: *p* < 0.001).

**Conclusions:**

A single session of prefrontal tACS significantly reduces the incidence of PSD on POD 1 and may offer additional benefits in reducing early postoperative anxiety and pain, with a favorable safety and tolerability profile.

**Trial Registration:**

China Clinical Trial Registration Center: ChiCTR2300078658

## Graphical abstract

A single preventive session of prefrontal tACS significantly reduces the incidence of PSD on the first postoperative day in patients undergoing gynecological laparoscopic surgery.

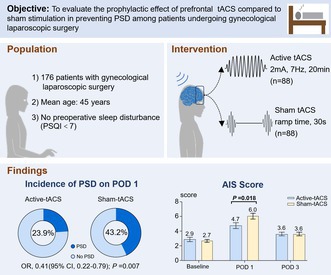

## 1 Introduction

Postoperative sleep disturbance (PSD), a common complication after surgery characterized by disrupted sleep patterns, poor sleep quality, and increased wakefulness, is often influenced by factors like pain, anxiety, and the effects of anesthesia [1]. The complex interplay of physiological, psychological, and environmental factors has led to a rising prevalence of PSD [1, 2]. PSD significantly contributes to severe postoperative complications, including heightened pain, delirium, and cardiovascular events, all of which can adversely impact patients' long‐term outcomes [3, 4, 5]. While pharmacological treatments like dexmedetomidine, zolpidem, and melatonin are commonly used as initial interventions, their use is limited by significant side effects, including dependency, circulatory issues, and respiratory depression [2, 6]. Given these limitations, the focus has shifted to non‐pharmacological approaches, such as cognitive‐behavioral therapy and music therapy, for managing PSD. However, the effectiveness of these methods largely depends on patients' willingness and can vary significantly between individuals [7]. Therefore, there is an urgent need for new, highly effective, well‐tolerated, and scalable therapies to significantly improve PSD patients' well‐being.

Recent research has explored the potential of non‐invasive brain stimulation techniques, particularly transcranial magnetic stimulation (TMS) and transcranial electrical stimulation (tES), in managing sleep disorders [8]. Lanza et al. [9] demonstrated that low‐frequency repetitive TMS, applied bilaterally to the dorsolateral prefrontal cortex, can effectively alleviate insomnia symptoms. However, while promising, this approach carries a risk of inducing seizures [10]. In contrast, tES provides a safer option by applying mild electrical currents to specific cortical areas via scalp electrodes to modulate neural activity [11, 12], exhibiting a superior safety profile compared to TMS [13]. Among tES methods, transcranial alternating current stimulation (tACS) stands out by its ability to synchronize with the brain's natural neural rhythms, allowing for more precise modulation of neural networks with minimal side effects [14, 15, 16]. Research indicates that tACS can enhance slow‐wave oscillations and increase the occurrence of sleep spindles during NREM sleep, thereby optimizing sleep architecture and prolonging sleep duration in both healthy individuals and insomnia patients [14]. Additionally, tACS has shown promise in addressing cognitive decline, anxiety, and depression associated with PSD, suggesting its potential as a transformative treatment for PSD [17, 18]. However, the prophylactic use of tACS to prevent insomnia during the perioperative period remains unexplored.

Therefore, this study employed a double‐blind design to compare the efficacy of tACS with sham control in the prophylactic treatment of PSD in patients undergoing gynecological laparoscopic surgery. We hypothesize that prefrontal tACS will reduce the incidence of PSD in this patient population.

## 2 Methods

### 2.1 Study Design

This study is a prospective, single‐center, randomized, double‐blind, sham‐controlled clinical trial, approved by the Institutional Scientific Research and Clinical Trials Ethics Committee of the First Affiliated Hospital of Zhengzhou University and registered on chictr.org.cn (ChiCTR2300078658). The manuscript adheres to the applicable CONSORT guidelines. Written informed consent was obtained from all participants or their legal surrogates prior to enrollment.

### 2.2 Study Population

All participants in the trial were thoroughly informed about the study protocol before their enrollment. Patients aged 18–65 years with American Society of Anesthesiologists (ASA) Class I to III, scheduled for gynecological laparoscopic surgery at the First Affiliated Hospital of Zhengzhou University in China between December 2023 and June 2024, were eligible for inclusion.

The exclusion criteria were as follows: (1) Preoperative Pittsburgh Sleep Quality Index (PSQI) higher than 7. (2) Contraindications to transcranial electrical stimulation, including the presence of electronic implants such as pacemakers or other metal devices, skull cavity or fractures, or local skin injury or inflammation. (3) History of neurosurgery, epilepsy, or mental disorders. (4) Long‐term use of sedative sleeping pills. (5) Pregnant or lactation. (6) Body mass index (BMI) greater than 30 (calculated as weight in kilograms divided by height in meters squared). (7) Allergies to any required drugs in the study or inability to use a patient‐controlled IV analgesia pump. (8) Patient refusal to participate in the study.

### 2.3 Randomization and Blinding

Patients were centrally randomized in a 1:1 ratio to either the active‐tACS or sham‐tACS group using a computer‐generated randomization table. The allocation information was placed in sealed envelopes, and a tACS operator who was not involved in the study administered the tACS treatment according to the allocation upon the patient's arrival at the post‐anesthesia care unit (PACU). The investigators (N.D., Jin Jin Y.) who were blinded to the treatment allocation monitored the patients in the intervention. All patients, anesthesiologists, other health care team members, and investigators who were responsible for data collection and follow‐ups were blinded to group allocation.

### 2.4 tACS Procedure

After surgery, tACS was administered to patients immediately after extubation in the PACU. The electrical stimulation was delivered through two electrodes embedded in conductive paste and securely fastened with an elastic cap. The electrodes were positioned according to the Fp1 and Fp2 locations specified in the international 10/20 electrode placement system, targeting the prefrontal cortex (PFC) region of the brain. The equipment used in this trial was provided by Neuracle Technology Co. (Changzhou, Jiangsu Province, China).

Patients in the active‐tACS group received a single 20‐min session of tACS immediately after extubation, using a frequency of 7 Hz and an intensity of 2 mA. The session began with a 30‐s ramp‐up phase to gradually increase the stimulation and ended with a 30‐s ramp‐down phase for a smooth cessation. In contrast, patients in the sham‐tACS group underwent a similar protocol, including the initial and final 30‐s ramp‐up and ramp‐down phases, but without the actual 2 mA alternating current stimulation during the 20‐min session.

Adverse events at the stimulation site were assessed through patient self‐reporting. Reported symptoms were categorized as “tingling”, “burning” or “other” intensity was rated as “mild”, “moderate”, or “severe”; and duration was qualitatively recorded as “transient” (resolving within 5 min) or “prolonged”. If a patient reported unbearable local discomfort, the stimulation was immediately terminated, and the event was recorded.

### 2.5 Anesthesia Procedure

Routine intraoperative monitoring was established upon the patient's arrival in the operating room, including noninvasive blood pressure measurement, pulse oximetry, and electrocardiography. Anesthesia was induced using 0.2 mg/kg etomidate, 0.2–0.3 μg/kg sufentanil, 0.2 mg/kg cisatracurium, 0.3 mg penehyclidine, and 10 mg dexamethasone. Once an adequate depth of anesthesia was achieved, the tracheal catheter was inserted.

Anesthesia was maintained with a continuous infusion of remifentanil (0.1–0.3 μg·kg^−1^ ·min^−1^), propofol (2–4 mg·kg^−1^·h^−1^), and sevoflurane at 0.3 MAC after age adjustment, along with intermittent injections of cisatracurium (2–4 mg). The infusion rate of anesthetics was titrated to keep the bispectral index values within the range of 40–60. Vasoactive drugs were used as needed to maintain blood pressure fluctuations within 20% of the baseline. Approximately 20 min before the end of surgery, the anesthesiologist administered 0.15 μg/kg of sufentanil and 50 mg of flurbiprofen axetil intravenously for postoperative analgesia. To prevent postoperative nausea and vomiting, 0.25 mg of palonosetron was also administered. After the surgery was completed, the tracheal catheter was removed once the patient regained sufficient muscle strength and was able to follow verbal commands. Oxycodone (5 mg) was provided as a rescue analgesic if the NRS pain score in the PACU exceeded 4 points. Postoperative pain was managed using a patient‐controlled intravenous analgesia (PCIA) pump, which included 0.2 mg/kg of hydromorphone, 100 mg of flurbiprofen axetil, 0.25 mg of palonosetron, and saline to a total volume of 200 mL. The PCIA pump was set to deliver a background infusion of 2.5 mL/h, with a bolus of 3 mL and a lockout time of 15 min. The use of the PCIA pump was dynamically managed based on the patient's pain levels to minimize the impact of pain on postoperative sleep.

### 2.6 Outcome Measures

The primary outcome was the incidence of PSD on postoperative day (POD) 1. Sleep quality was assessed using the Athens Insomnia Scale (AIS) on preoperative day 1, and on PODs 1 and 3. PSD was defined as an AIS score of 6 points or higher. The secondary outcomes included: (1) The incidence of PSD on POD 3. (2) Changes in other sleep parameters measured via the AIS, including total score, sleep onset latency (SOL), night awakening score (NAS), final awakening, total sleep time (TST), sleep quality, and daily disturbances (represented by the sum of the scores on the 6th, 7th, and 8th items of the AIS). (3) Anxiety and depression scores on PODs 1 and 3, measured using the Hospital Anxiety and Depression Scale (HADS). (4) Postoperative pain scores at rest and during movement at 24 and 72 h, measured using the numeric rating scale (NRS), as well as postoperative consumption of hydromorphone at 24 and 72 h. (5) Incidence of adverse events, including nausea and vomiting, headache and dizziness, discomfort at the site of electrical stimulation, nightmares, and recovery quality as measured by the Quality of Recovery‐15 (QoR‐15).

AIS is an internationally recognized, reliable self‐assessment tool for measuring the severity of sleep difficulties based on the International Classification of Diseases, 10th revision (ICD‐10). The AIS includes 8 items: the first five assess sleep‐related complaints (sleep induction, waking up at night, early morning awakening, total sleep time, and overall quality of sleep), and the last three items assess the consequences of insomnia on daily functioning (well‐being, functional ability, and daytime sleepiness). The AIS score ranges from 0 to 24 points, with a score of 6 or higher indicating insomnia.

The NRS score ranges from 0 (indicating no pain) to 10 (indicating intolerable pain) points. The HADS consists of 14 questions, with 7 items each for the anxiety and depression subscales. The score for each item ranges from 0 to 3 points, and a total score of 8 points or higher is diagnosed as depression or anxiety. The QoR‐15 scale includes 15 questions, with a total score of 150 points. A final score of 118 or higher indicates good postoperative recovery.

To evaluate the safety and feasibility of the experiment, researchers recorded the occurrence of adverse reactions and provided appropriate treatment. Additionally, data on surgery and anesthesia duration, consumption of intraoperative remifentanil and propofol, estimated infusion volume and blood loss, transfusion status, tracheal catheter removal time (time from the end of surgery to removal of the tracheal catheter), and length of hospital stay were recorded.

### 2.7 Sample Size Calculation

The sample size was determined using PASS 15.0 software. Preliminary data indicated a 40% incidence rate of PSD among patients undergoing gynecological laparoscopic surgery on POD 1. We hypothesized that reducing the incidence of PSD in the intervention group to 20% would be considered effective [19]. A study power of 0.80 and a significance level of 0.05 were set, using a two‐sided test to detect statistically significant differences in PSD incidence. Based on these parameters, a minimum of 79 patients per group was required. To account for a potential 10% loss to follow‐up, the sample size was conservatively increased to 88 patients per group to ensure robustness and reliability in the study outcomes.

### 2.8 Statistical Analysis

Statistical analysis was performed using SPSS, version 25.0 (IBM SPSS). Continuous data with a normal distribution were represented by mean and standard deviation (SD) and analyzed using independent sample *t*‐tests. Non‐normally distributed data were represented by median and interquartile range (IQR) and analyzed using Mann–Whitney *U* tests. The normality of the variables was assessed using the Kolmogorov–Smirnov test. Categorical variables were reported as number (%) and compared using χ^2^ tests or Fisher's exact test. Differences in dichotomous outcomes were described using 95% confidence intervals (CIs).

To assess selection bias and result robustness, both intention‐to‐treat (ITT) and per‐protocol (PP) analyses were conducted for baseline characteristics and the primary outcome (incidence of PSD on POD 1). The primary outcome was analyzed using the χ^2^ test, with missing data handled by multiple imputation (5 imputed datasets generated; imputation models included baseline BMI, ASA class, age, surgery type, PSQI, and HADS‐A/D scores, with final datasets averaged). For secondary outcomes, while subgroup analyses used ITT, other measures adopted PP analysis (missing cases excluded directly). Repeated‐measures data were analyzed via generalized estimating equations (GEE) to assess treatment‐by‐time interactions, with Bonferroni corrections for multiple comparisons when interactions were significant. A two‐sided *p* < 0.05 was considered significant.

To gain deeper insights into the primary outcome, exploratory analyses were conducted to evaluate differences within subgroups based on age, ASA classification, surgery type, anemia status, history of insomnia, preoperative AIS score, and preoperative HADS‐A score. The interaction between these variables and treatment was assessed using binary logistic regression. The mediation model was analyzed using Model 4 in the PROCESS Macro. For the best test of the mediation effect, a bootstrapping procedure was used to measure the indirect effect, with 95% CIs estimated. If the confidence interval included zero, it indicated no significant mediating effect at a significance level of 5%.

## 3 Results

### 3.1 Flowchart of the Study

**FIGURE 1:**
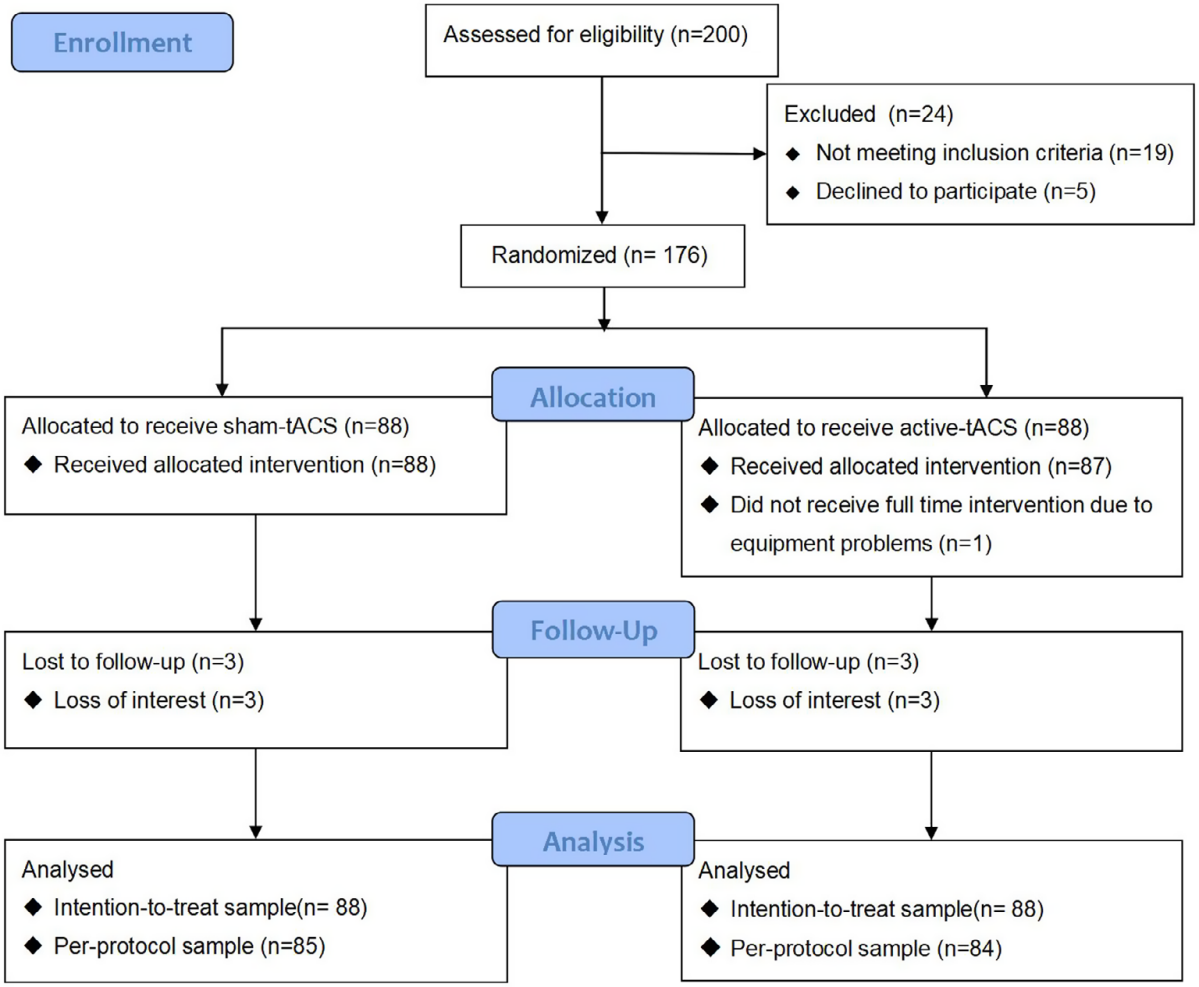
Flowchart of the study. TACS, transcranial alternating current stimulation.

A total of 200 patients were screened for inclusion from December 2023. Of these, 24 patients were excluded based on the exclusion criteria, including 5 who refused to participate and 19 who did not meet the inclusion criteria. Consequently, 176 patients were recruited and randomly assigned to the active‐tACS group and the sham‐tACS group. After randomization, one patient in the active‐tACS group did not receive the full intervention due to equipment problems. During the three‐day postoperative follow‐up, three patients in each group were lost to follow‐up. Ultimately, 169 patients completed the trial, with 84 in the active‐tACS group and 85 in the sham‐tACS group. The participant flow diagram is shown in Figure 1.

### 3.2 Baseline Demographics and Clinical Characteristics

The analysis of the 176 patients revealed comparable demographic profiles between the two groups (Table 1). There were no significant differences in preoperative anxiety and depression scores (using HADS), sleep quality scores (using PSQI and AIS), or the proportion of patients with a history of insomnia (7 patients in total, 4 in the active‐tACS group and 3 in the sham‐tACS group; *p* > 0.05).

**TABLE 1:** Baseline characteristics of patients allocated to active‐tACS group or sham‐tACS group.^a^

Characteristic	Participants, No. (%)		*p*
	Active‐tACS (*n* = 88)	Sham‐tACS (*n* = 88)	
Age, median (IQR), y	45.0 (40–51)	47.5 (40–53)	0.171
Height, median (IQR), cm	160 (158–163)	160 (157–164)	0.409
BMI, mean (SD), kg m^−2^	24.1 (3.13)	24.4 (3.07)	0.481
ASA classification			0.533
I	57 (64.8)	53 (60.2)	
II	31 (35.2)	35 (39.8)	
Surgery type			0.646
Ovarian cystectomy	20 (22.7)	20 (22.7)	
Myomectomy	19 (21.6)	14 (15.9)	
Hysterectomy	42 (47.7)	43 (48.9)	
Radical resection of endometrial cancer	7 (8.0)	11 (12.5)	
Comorbidities			0.686
Diabetes	5 (5.7)	6 (6.8)	
Hypertension	16 (18.2)	19 (21.6)	
Coronary artery disease	1 (1.1)	3 (3.4)	
Anemia	16 (18.2)	13 (14.8)	0.542
History of insomnia	4 (4.6)	3 (3.4)	1.000
Smoking	1 (1.1)	1 (1.1)	1.000
Alcohol abuse	0	0	—
HADS‐A score, median (IQR)	5 (4–5)	5 (3–5.8)	0.399
HADS‐D score, median (IQR)	2 (2–3)	2 (2–3)	0.580
AIS, median (IQR)	2 (1–4)	2 (1–4)	0.751
PSQI, median (IQR)	3 (2–4)	2 (1.8–3)	0.075

Abbreviations: AIS, Athens Insomnia Scale; ASA, American Society of Anesthesiologists physical status classification; BMI, body mass index (calculated as weight in kilograms divided by height in meters squared); HADS‐A, Hospital Anxiety and Depression Scale‐Anxiety; HADS‐D, Hospital Anxiety and Depression Scale‐Depression; PSQI, Pittsburgh Sleep Quality Index; tACS, transcranial alternating current stimulation.

^a^ Data presented as median (IQR) were compared using the Mann–Whitney test. Data presented as mean (SD) were compared using the unpaired, 2‐tailed *t* test. Data reported as the number of patients (%) were compared using the Pearson χ^2^.

To assess potential selection bias, we compared baseline characteristics between the ITT and PP populations. As detailed in Table S1, all baseline variables were similar across groups (*p* > 0.05; Table S1 in Supporting Information), indicating that the PP population is representative of the ITT population and strengthening the validity of our findings.

### 3.3 Primary Outcome

**FIGURE 2:**
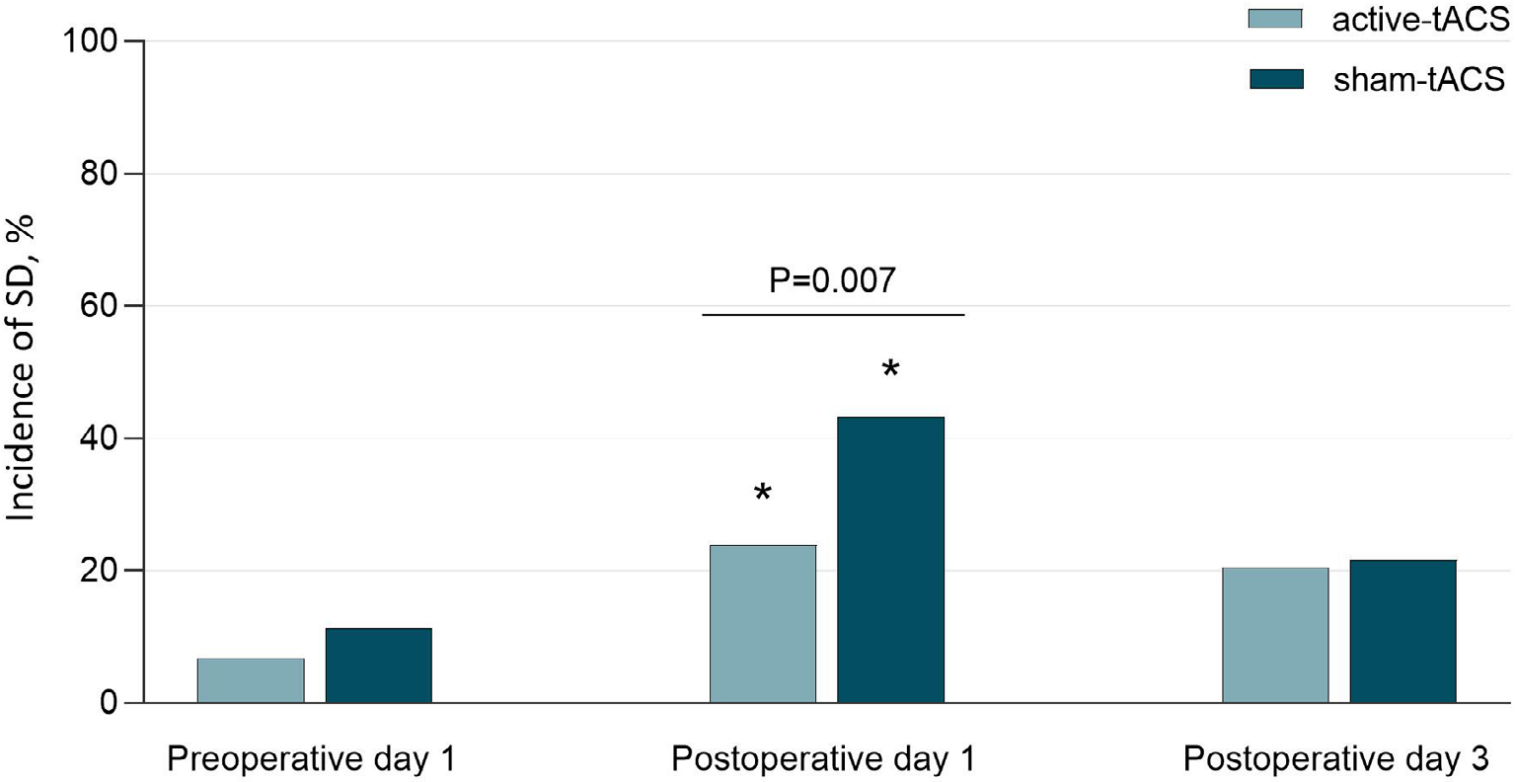
Incidence of Sleep Disturbance (SD) on Preoperative Day 1, Postoperative Day 1, and Postoperative Day 3. Data on the intention‐to‐treat sample were analyzed using the Pearson χ^2^. Intergroup difference in sleep disturbance incidence on postoperative day 1 (Active vs. Sham): *P* = 0.007. *Compared to baseline sleep disturbance incidence (preoperative day 1): *p* < 0.01.

In line with the ITT principle, the primary outcome analysis showed that the incidence of PSD on POD 1 was significantly lower in the active‐tACS group (23.9%) than in the sham‐tACS group (43.2%) (OR, 0.41 [95% CI, 0.22–0.79]; *p* = 0.007; Figure 2; Table S2 in Supporting Information). Furthermore, both groups exhibited a statistically significant increase in PSD incidence on POD 1 compared to their preoperative baselines (Figure 2).

As a sensitivity analysis, PP analysis of the primary outcome was also conducted. The overall incidence of PSD in the PP population on POD 1 was 33.7% (57 of 169 patients). Results showed that the PSD incidence on POD 1 was 23.8% in the active‐tACS group and 43.5% in the sham‐tACS group (OR, 0.41 [95% CI, 0.21–0.79]; *p* = 0.007; Table S2 in Supporting Information), consistent with the ITT analysis findings. These consistent results strengthen the robustness of our conclusions.

### 3.4 Secondary Outcomes

In the ITT analysis, there was no significant difference in the incidence of PSD on POD 3 between the two groups (20.5% vs. 21.6%; OR, 0.93 [95% CI, 0.45–1.93]; *p* = 0.853), consistent with the PP analysis results (*p* = 0.884) (Table S2 in Supporting Information). Regarding sleep parameters measured by the AIS, a significant interaction between time and group was observed. Specifically, the AIS scores in the active‐tACS group showed a statistically significant reduction on POD 1 compared to the sham‐tACS group (B = −1.29; 95% CI, −2.35 to −0.22; *p* = 0.018; Table 2). The improvement in sleep was mainly reflected by increased TST, better sleep quality, and reduced daily disturbances (*p* < 0.050).

**TABLE 2:** Sleep quality on PODs 1 and 3 in the active‐tACS group or sham‐tACS group.^a^

Characteristic	Participants, No. (%)		B	*p*
	Active‐tACS (*n* = 84)	Sham‐tACS (*n* = 85)		
POD1				
AIS, median (IQR)	4 (3–5)	5 (3–8)	−1.285	0.018
SOL, median (IQR)	0 (0–1)	0 (0–1)	−0.161	0.153
NAS, median (IQR)	1 (1–2)	1 (0–2)	0.097	0.500
Final awaking, median (IQR)	0 (0–1)	1 (0–1)	−0.146	0.174
TST, median (IQR)	0 (0–1)	1 (0–1)	−0.311	0.015
Sleep quality, median (IQR)	0 (0–0.3)	0 (0–1)	−0.243	0.042
Daily disturbances,^b^ median (IQR)	2 (1–2)	2 (2–3)	−0.485	0.003
POD3				
AIS, median (IQR)	2 (2–5)	3 (2–5)	0.089	0.817
SOL, median (IQR)	0 (0–1)	0 (0–1)	−0.067	0.394
NAS, median (IQR)	1 (1–1)	1 (0–1)	0.224	0.050
Final awaking, median (IQR)	0 (0–1)	0 (0–1)	−0.018	0.832
TST, median (IQR)	0 (0–1)	0 (0–1)	0.005	0.959
Sleep quality, median (IQR)	0 (0–0)	0 (0–1)	−0.056	0.459
Daily disturbances,^b^ median (IQR)	1 (1, 1)	1 (1, 1)	−0.068	0.534

Abbreviations: AIS, Athens Insomnia Scale; NSA, night awakening score; PSD, postoperative sleep disturbance; SOL, sleep onset latency; tACS, transcranial alternating current stimulation; TST, total sleep time.

^a^ Data were analyzed using the per‐protocol sample. Data presented as median (IQR) were compared using generalized estimating equation (GEE) analysis. Data reported as the number of patients (%) were compared using the Pearson χ^2^.

^b^ Represented by the sum of scores for well‐being, functional ability, and daytime sleepiness in the AIS.

**FIGURE 3:**
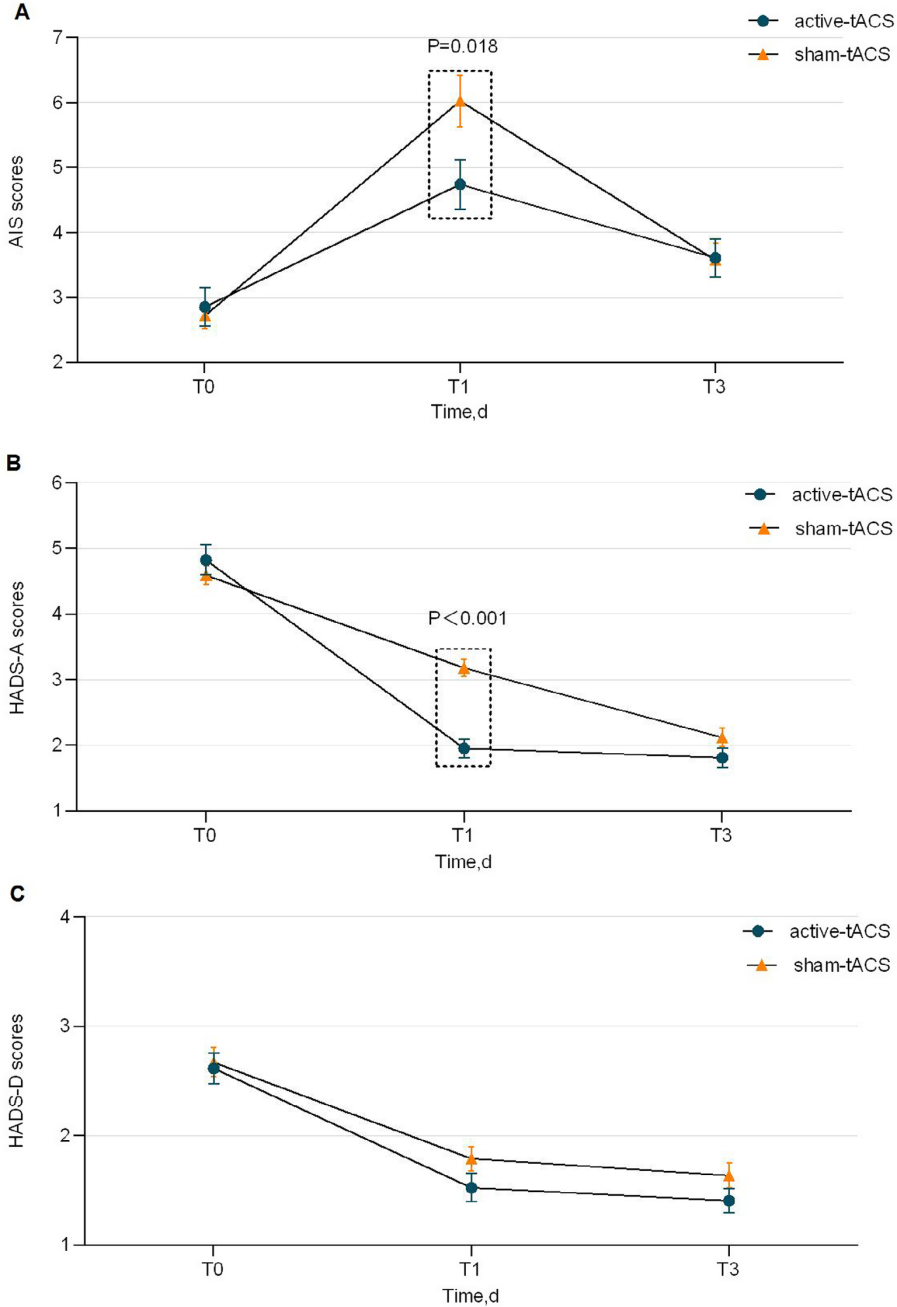
Comparison of AIS, HADS‐A, and HADS‐D scores between the two groups. Error bars represent the SE of the mean. Data on the per‐protocol sample were analyzed using Generalized estimating equation (GEE) analysis. (A): Changes in AIS scores over time. Intergroup difference at T1 (Active vs. Sham): *p* = 0.018. (B): Changes in HADS‐A scores over time. Intergroup difference at T1 (Active vs. Sham): *p* < 0.001. (C): Changes in HADS‐D scores over time. Abbreviations: T0, preoperative day 1; T1, postoperative day 1; T3, postoperative day 3.

Both groups initially showed an increase in AIS scores followed by a decrease, but the AIS scores in the sham‐tACS group remained significantly higher than baseline on POD 3 (*p* = 0.050) (Table 2; Figure 3A; Table S3 in Supporting Information).

The GEE analysis revealed that anxiety and depression levels, as measured by the HADS, progressively decreased over time in both groups, with statistically significant reductions compared to preoperative levels (*p* < 0.001). The intergroup analysis showed that anxiety scores (HADS‐A) differed significantly between the two groups only on POD 1 (B = ‐1.22; *p* < 0.000), while depression scores (HADS‐D) did not show significant differences between the groups throughout the study period (*p* = 0.228) (Figure 3B; Figure 3C; Table S3 in Supporting Information).

Notably, the active‐tACS group reported significantly lower pain scores, both at rest and during movement, on POD 1 (movement: *p* = 0.002; rest: *p* < 0.001) and POD 3 (movement: *p* = 0.028; rest: *p* < 0.001). However, there were no significant differences in the rescue analgesia rate of oxycodone postoperatively, median (IQR) hydromorphone consumption during the first 24 h after surgery (3.6 [2.3–4.0] mg vs. 3.5 [2.6–4.1] mg; *p* = 0.767) or total consumption 72 h after surgery (6.0 [4.9–8.2] mg vs. 6.4 [5.1–7.5] mg; *p* = 0.502) (Table S4 in Supporting Information; Table 3).

**TABLE 3:** Comparison of pain scores and opioid consumption between the two groups on PODs 1 and 3.^a^

Variables	Active‐tACS (*n* = 84)	Sham‐tACS (*n* = 85)	*p*
POD1			
NRS scores for pain at motion, median (IQR)	3 (3–4)	4 (3–4)	0.002
NRS scores for pain at rest, median (IQR)	1 (1–2)	2 (1–3)	< 0.001
Hydromorphone consumption, median (IQR), mg	3.6 (2.3, 4.0)	3.5 (2.6, 4.1)	0.767
POD3			
NRS scores for pain at motion, median (IQR)	2 (2–3)	2 (2–3)	0.028
NRS scores for pain at rest, median (IQR)	1 (0–1)	1 (1–2)	< 0.001
Hydromorphone consumption, median (IQR), mg	6.0 (4.9, 8.2)	6.4 (5.1, 7.5)	0.502

Abbreviation: NRS, numeric rating scale.

^a^ Data were analyzed using the per‐protocol sample. Data presented as median (IQR) were compared using the Mann–Whitney test.

Furthermore, no significant differences were observed between the active‐tACS and sham‐tACS groups in terms of surgery and anesthesia duration, intraoperative consumption of remifentanil and propofol, estimated infusion volume, blood loss, transfusion requirements, extubation time, or length of hospitalization. Additionally, there was no significant difference in the QoR‐15 score on POD 3 between the two groups (130 [127–133] vs. 129 [126.5–131] points; *p* = 0.114; Table S4 in Supporting Information).

Two patients in the active‐tACS group experienced slight skin tingling at the site of electrical stimulation, which they reported as transient and tolerable, but no significant differences were observed between the two groups. Similarly, the incidence of other adverse events, such as nausea and vomiting, headache and dizziness, and nightmares, did not significantly differ between the two groups (Table 4).

**TABLE 4:** Incidence of adverse events in the active‐tACS group or sham‐tACS group in the trial.^a^

Characteristic	Participants, No. (%)		*p*
	Active‐tACS (*n* = 84)	Sham‐tACS (*n* = 85)	
Nausea and vomiting	24 (28.6)	27 (31.8)	0.651
Headache and dizziness	20 (23.8)	20 (23.5)	0.966
Discomfort at the site of electrical stimulation	2 (2.4)	0	0.246
Nightmare	0	0	1.000

^a^ Data presented as the number of patients (%) were compared using the Pearson χ^2^ test or Fisher exact test.

**FIGURE 4:**
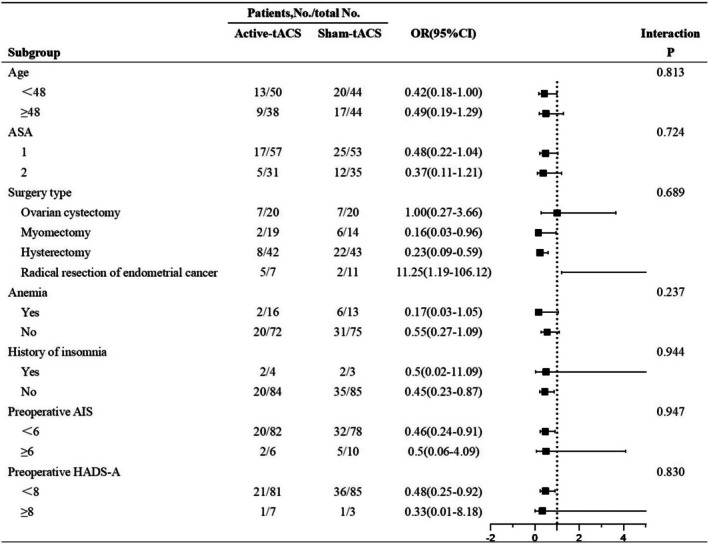
Forest Plot of the Subgroup Analysis. AIS, Athens Insomnia Scale; HADS‐A indicates Hospital Anxiety and Depression Scale‐Anxiety; OR, odds ratio.

In exploratory analyses, the interaction term of the subgroup analysis was used as a statistical test to evaluate the impact of the intervention, with the results visually presented in the forest plot in Figure 4. The effect of tACS on the incidence of PSD on POD 1 was consistent across all seven subgroups (*p* > 0.05). However, given the limited sample size in some subgroups (*n* < 10), the results may exhibit substantial variability, so the interpretation of the conclusions should be cautious. To further explore whether tACS indirectly affects PSD by relieving pain or anxiety, we conducted a mediation analysis. The resting and movement pain scores (NRS) and anxiety scores (HADS‐A) were treated as mediator variables and incorporated into the mediation model. The results showed no significant mediating effects for any of the three variables (Table S5 in Supporting Information).

## 4 Discussion

This prospective, randomized controlled trial highlights the prophylactic benefits of a single postoperative tACS session targeting the prefrontal cortex in reducing the incidence of PSD in patients undergoing gynecological laparoscopic surgery. Additionally, this innovative therapy shows potential for reducing early postoperative anxiety and alleviating postoperative pain, all while maintaining excellent safety and tolerability profiles.

Existing research shows PSD incidence up to 42% in general surgery patients, with 23% persisting until the fourth postoperative day [20]. Gynecological surgery patients experience higher rates (31.4% to 78%), especially among perimenopausal women and those undergoing tumor resection [21, 22, 23]. Our trial reflects these findings, with a similar PSD incidence observed (43.5% in the control group on POD 1; 22.4% on POD 3). Our results also indicate that PSD incidence peaks on POD 1 in both groups, gradually declining to preoperative levels by POD 3. This pattern of initial deterioration followed by improvement aligns with previous studies, including studies by Gu et al. on postoperative sleep quality in gynecological patients [24], and a meta‐analysis highlighting a significant reduction in REM sleep post‐surgery, with recovery over the following four days [25]. This improvement in postoperative sleep quality may be attributed to reduced surgical stress response, effective postoperative pain management, and anesthetic effects diminishing.

The PACU stage is a critical period during which patients transition from anesthesia to wakefulness. Intervention at this stage may be more conducive to targeting the specific mechanisms of PSD, such as regulating disordered neurotransmitter levels and neural network activity. Additionally, intervening in the PACU allows for preemptive regulation of cortical neural activity before patients enter natural sleep, making it easier for them to enter a normal sleep cycle. Our intergroup analysis highlights a significant improvement in postoperative sleep quality on POD 1, with tACS applied in PACU effectively reducing the incidence of PSD by half on this day. Notably, the 7 Hz frequency aligns with the frontopolar cortex's intrinsic theta oscillations. Within this band, single stimulation facilitates sleep onset and prolongs slow‐wave sleep [26, 27], mechanistically involving tACS's ability to regulate brainwave rhythms and correct abnormal brain network activities during sleep [28]. Although our findings did not show a significant difference in sleep quality between the groups on POD 3, the intragroup comparison revealed distinct recovery patterns: sleep quality scores in the active‐tACS group returned to baseline by POD 3, whereas the sham‐tACS group remained below baseline levels. This divergence suggests a potential early restorative effect of tACS. However, the lack of day 2 data limits our ability to characterize the time‐dependent trajectory of this intervention. Building on this evidence, our findings further suggest that tACS may serve as a promising intervention for alleviating acute PSD.

While our findings demonstrate the acute benefits of a single tACS session, we acknowledge that the treatment of patients with persistent PSD is equally crucial. Evidence suggests that repeated interventions may amplify and prolong therapeutic benefits. Wang et al. [29] demonstrated that 20 tACS sessions in patients with insomnia resulted in significant therapeutic benefits that lasted for at least 8 weeks. Moreover, repetitive low‐frequency tACS over the frontal cortex promotes neuroplasticity and provides long‐term stability, as evidenced by increased theta and beta power in EEG, strengthened functional connectivity between the frontal and parietal cortices, enhanced interhemispheric connectivity, and elevated oxyhemoglobin levels in the frontal cortex [30]. Despite the significant challenges associated with repeated tACS treatment during the perioperative period (e.g., patient compliance limitations), developing neuromodulation strategies involving repetitive stimulation to sustain long‐term therapeutic efficacy remains a critical direction for future research.

Sleep disturbances often coexist with mood disorders, exhibiting a bidirectional relationship in which each can exacerbate the other. Clinically, about 70% of individuals diagnosed with anxiety and depression struggle with sleep issues [31, 32], while approximately 30% of patients with sleep disorders also suffer from anxiety [33]. Despite this, effectively addressing comorbid sleep disorders, anxiety, and depression during the perioperative period remains a significant challenge. Recent studies have shed light on the neural mechanisms underlying this complex interplay, highlighting the role of functional connectivity among key brain regions—such as the lateral orbitofrontal cortex, dorsolateral prefrontal cortex, precuneus, amygdala, and insula—in modulating the sleep‐anxiety‐depression nexus [34, 35]. The prefrontal region targeted by the FP1/FP2 sites exerts a direct influence on improving sleep quality [36] and is also implicated in emotional regulation [37] and stress response [38]. Our study confirms these insights, demonstrating that tACS applied at the FP1/FP2 sites can not only mitigate PSD but also effectively reduce early postoperative anxiety. This finding is consistent with reports highlighting the ability of tACS to modulate endogenous brain oscillations and influence emotional processing [39, 40, 41, 42]. However, tACS did not significantly improve postoperative depression, which may be due to the relatively low baseline depression scores among our study participants, suggesting a potential floor effect. Overall, our findings highlight the potential of tACS as an effective intervention for preventing and managing perioperative sleep disorders, along with their associated anxiety and depression symptoms, offering a promising avenue for future clinical practice.

Interestingly, our study revealed an unexpected outcome, with pain management showing more favorable results than sleep improvement. Previous research has highlighted the complex role of the prefrontal cortex in both acute and chronic pain processes, where changes in neurotransmitters, gene expression, glial cell activity, and neuroinflammation contribute to pain modulation, either relieving or amplifying it [43]. Our findings demonstrate a sustained analgesic effect of tACS on postoperative pain, consistent with earlier studies that emphasize the effectiveness of prefrontal cortex tACS in reducing subjective pain perception [44]. Although statistically significant improvements in pain scores were observed, no intergroup difference was found in opioid consumption. This result may suggest that the magnitude of pain relief from the current intervention has not reached the clinical threshold for adjusting drug dosages, or that the mechanism of tACS tends to modulate the emotional dimension of pain rather than the intensity of nociceptive signals [45]. It is noteworthy that most patients in this study had low pain scores and required minimal opioids, which may have rendered the dosage range insufficiently sensitive to detect differences. While the numerical difference in pain scores between the two groups on postoperative days 1 and 3 was minimal and did not reach the MCID, a more detailed analysis revealed a significant reduction in the incidence of moderate to severe postoperative pain in the active‐tACS group compared to the sham‐tACS group (31% vs. 58.8%; OR, 0.31 [95% CI, 0.17–0.59]; *p* < 0.001; Table S6 in Supporting Information). This finding suggests that the specific tACS parameters used in our study may be effective in reducing the incidence of severe postoperative pain, highlighting tACS as a promising intervention for postoperative pain management. However, further refinement of stimulation parameters and targeting of specific brain regions is necessary to optimize its effectiveness.

Moreover, despite the well‐established link between pain relief, mood enhancement, and improved sleep quality [1, 46], our analysis of mediating factors suggests that tACS directly contributed to the reduction in PSD incidence in this trial, rather than through the alleviation of anxiety and pain. This observation underscores the multifaceted and direct impact of tACS on postoperative recovery, emphasizing the need for further investigation into its precise mechanisms and potential for broader therapeutic applications.

This study acknowledges several limitations that warrant consideration. First, it is a single‐center study with an experimental population limited to gynecological women. The variability in treatment approaches and clinical practices across different patient groups and medical centers could affect the external validity and generalizability of the results. Therefore, it is necessary to conduct future research in a broader range of populations and multi‐center settings, thereby enhancing the universal applicability of the findings. Second, although data collection covered postoperative days 1 and 3, providing critical evidence supporting the postoperative application of tACS, the lack of extended follow‐up may underestimate the true burden of sleep disruption and prevent differentiation between short‐term and sustained effects of tACS. Future studies should validate long‐term benefits and translational potential by extending follow‐up periods. Third, while we have demonstrated the efficacy of the specific stimulation parameters used in this trial, the search for an optimal stimulation regimen—including variations in sites, frequencies, and current intensities—remains an open question that requires further investigation. Fourth, although this study ensured the effectiveness of blinding through measures such as consistent device appearance, standardized operation procedures, and sham stimulation simulation, the lack of direct assessment of participants' blinding status remains one of the limitations of this study. Finally, the study's reliance on subjective measures limits our understanding of tACS's impact on postoperative sleep. Incorporating objective indicators, such as polysomnography or biomarkers, would provide a more comprehensive assessment of tACS's effects. Additionally, integrating tACS with neuroimaging techniques, such as EEG, functional MRI, and near‐infrared spectroscopy, could help elucidate the underlying neural mechanisms and structural or functional changes induced by tACS. This approach could potentially lead to the development of tailored treatment strategies that are better suited to individual patients' needs.

## 5 Conclusions

This randomized, double‐blind, sham‐controlled trial demonstrates that a single postoperative tACS session targeting the prefrontal cortex effectively reduces the incidence of PSD in patients undergoing gynecological laparoscopic surgery.

## Author Contributions

**Ni Du:** conceptualization, methodology, data curation, formal analysis, investigation, and writing – original draft, writing – review and editing. **Jin‐Jin Yang:** methodology, data curation, formal analysis, investigation. **Xin‐Yu Li** and **Zhi‐Hao Li:** data curation, formal analysis, investigation. **Kenji Hashimoto:** validation, writing – review and editing. **Lei Lei:** conceptualization, methodology, investigation, project administration, funding acquisition, writing – original draft, writing – review and editing. **Jian‐Jun Yang:** conceptualization, funding acquisition, supervision, and writing – review and editing.

## Ethics Statement

All procedures were approved by the Institutional Scientific Research and Clinical Trials Ethics Committee of the First Affiliated Hospital of Zhengzhou University, ensuring compliance with the ethical standards and regulations of the Declaration of Helsinki on Human Research.

## Consent

All participants were fully informed of the study procedure and voluntarily signed a written informed consent form.

## Conflicts of Interest

The authors declare no conflicts of interest.

## Supporting information


**Table S1.** Baseline characteristics comparison between ITT and PP populations.
**TABLE S2.** Incidence of Postoperative Sleep Disorder (PSD) by ITT and PP approaches.
**TABLE S3.** Generalized estimation equation (GEE) analysis of AIS, HADS‐A and HADS‐D.
**TABLE S4.** Intraoperative and postoperative data by the two groups.
**TABLE S5.** Mediating model examination by bootstrap.
**TABLE S6.** Incidence of mild, moderate and severe pain between the two groups.

## Data Availability

The data that support the findings of this study are available from the corresponding author upon reasonable request.
