# Supplementary material for: Prophylactic Effect of Prefrontal Alternating Current Stimulation on Postoperative Sleep Disturbance in Patients Undergoing Gynecological Laparoscope: A Randomized, Double‐Blind, Sham‐Controlled Trial
Source: CNS Neurosci Ther. 2025 Aug 4;31(8):e70529. doi: 10.1111/cns.70529 (PMC12320130; doi:10.1111/cns.70529)
Supplement: Supplementary file 1 — Table S1. Baseline characteristics comparison between ITT and PP populations. TABLE S2. Incidence of Postoperative Sleep Disorder (PSD) by ITT and PP approaches. TABLE S3. Generalized estimation equation (GEE) analysis of AIS, HADS‐A and HADS‐D. TABLE S4. Intraoperative and postoperative data by the two groups. TABLE S5. Mediating model examination by bootstrap. TABLE S6. Incidence of mild, moderate and severe pain between the two groups. [file CNS-31-e70529-s001.docx]

**supplementary material**

**etable1.** Baseline Characteristics Comparison Between ITT and PP Populations

**etable 2.** Incidence of Postoperative Sleep Disorder (PSD) by ITT and PP Approaches

**etable 3.** Generalized estimation equation (GEE) analysis of AIS, HADS-A and HADS-D

**etable 4.** Intraoperative and postoperative data by the two groups.

**etable 5.** Mediating model examination by bootstrap.

**etable 6.** Incidence of mild, moderate and severe pain between the two groups.

This supplemental material has been provided by the authors to give readers additional information about their work

**etable1.Baseline characteristics comparison between ITT and PP populations**

|  | **Participants, No. (%)** |  |  |
| --- | --- | --- | --- |
| **Baseline Characteristics** | **ITT Population**  **(n = 176)** | **PP Population**  **(n = 169)** | ***P* value** |
|  |  |  |  |
| Age, median (IQR), y | 47(40-52) | 47 (40-52) | 0.951 |
| Height, median (IQR), cm | 160 (158-164) | 160 (158-164) | 0.998 |
| BMI, mean (SD), kg m-2 | 24.2 (3.1) | 24.2 (3.1) | 0.980 |
| ASA classification |  |  | 0.854 |
| Ⅰ | 110(62.5) | 104 (61.5) |  |
| Ⅱ | 66(37.5) | 65 (38.5) |  |
| Surgery type |  |  | 0.999 |
| Ovarian cystectomy | 40 (22.7) | 38 (22.5) |  |
| Myomectomy | 33 (18.8) | 31 (18.3) |  |
| Hysterectomy | 85 (48.3) | 83 (49.1) |  |
| Radical resection of endometrial cancer | 18 (10.2) | 17 (10.1) |  |
| Comorbidities |  |  | 0.999 |
| Diabetes | 11 (6.3) | 11 (6.5) |  |
| Hypertension | 35 (19.9) | 34 (20.1) |  |
| Coronary artery disease | 4 (2.3) | 4(2.4) |  |
| Anemia | 29 (16.5) | 29 (17.2) | 0.865 |
| History of insomnia | 7 (4.0) | 7 (4.1) | 0.938 |
| Smoking | 2 (1.1) | 2 (1.2) | 1.000 |
| Alcohol abuse | 0 | 0 | - |
| HADS-A score, median (IQR) | 5 (4-5) | 5 (4-5) | 0.892 |
| HADS-D score, median (IQR) | 2 (2-3) | 2 (2-3) | 0.837 |
| AIS, median (IQR) | 2(1-4) | 2 (1-4) | 0.998 |
| PSQI, median (IQR) | 3 (2-4) | 3 (2-4) | 0.990 |

Data presented as median (IQR) were compared using the Mann-Whitney test. Data presented as mean (SD) were compared using the unpaired, 2-tailed t test. Data reported as the number of patients (%) were compared using the Pearson χ2.

Abbreviations:ITT, Intention-to-Treat ; PP, Per-Protocol,

**eTable 2.Incidence of Postoperative Sleep Disorder (PSD) by ITT and PP Approaches^a^**

|  | **Participants,**  **No. (%)** |  |  |  |
| --- | --- | --- | --- | --- |
| **Analysis Method** | **Active-tACS Group** | **Sham-tACS Group** | **OR (95% CI)** | ***P* value** |
| POD 1 |  |  |  |  |
| Intention-to-Treat (ITT) | 21(23.9) | 38 (43.2) | 0.41(0.22-0.79) | 0.007 |
| Per-Protocol (PP) | 20(23.8) | 37(43.5) | 0.41(0.21-0.79) | 0.007 |
| POD 3 |  |  |  |  |
| Intention-to-Treat (ITT) | 18(20.5) | 19(21.6) | 0.93(0.45-1.93) | 0.853 |
| Per-Protocol (PP) | 18(21.4) | 19(22.4) | 0.95(0.46-1.97) | 0.884 |

^a^ITT population includes all randomized patients; PP population excludes protocol violators and dropouts.PP analysis presented as sensitivity analysis. ITT is the primary analysis approach.

Data presented as the number of patients (%) were compared using the Pearsonχ2

Abbreviations: OR, Odds Ratio; CI, Confidence Interval

**etable 3.Generalized estimation equation (GEE) analysis of AIS, HADS-A and HADS-D**

**etable 3.1Effect of Model Test**

|  | **Group** | |  | **Time** | |  | **Group×Time** | |
| --- | --- | --- | --- | --- | --- | --- | --- | --- |
|  | **Wald x^2^** | **P** |  | **Wald x^2^** | **P** |  | **Wald x^2^** | **P** |
| AIS | 1.25 | 0.264 |  | 86.53 | 0.000 |  | 8.16 | 0.017 |
| HADS-A | 5.17 | 0.023 |  | 588.41 | 0.000 |  | 156.25 | 0.000 |
| HADS-D | 1.48 | 0.228 |  | 168.07 | 0.000 |  | 1.44 | 0.488 |

**etable 3.2 Between-group and Within-group Pairwise Comparisons Of AIS**

|  | **B** | **SE** | **Wald**  **95% CI** | **P** |
| --- | --- | --- | --- | --- |
| Active-tACS Group |  |  |  |  |
| T1-T0 | 1.89 | 0.36 | 1.03~2.74 | 0.000 |
| T3-T0 | 0.75 | 0.34 | -0.07~1.58 | 0.084 |
| T3-T1 | -1.13 | 0.28 | -1.79~-0.47 | 0.000 |
| Sham-tACS Group |  |  |  |  |
| T1-T0 | 3.31 | 0.44 | 2.26~4.36 | 0.000 |
| T3-T0 | 0.80 | 0.31 | 0.05~1.55 | 0.05 |
| T3-T1 | -2.51 | 0.44 | -3.56~-1.45 | 0.000 |
| Between-group |  |  |  |  |
| T0(G1-G0) | 0.136 | 0.35 | -0.55~0.82 | 0.697 |
| T1(G1-G0) | -1.285 | 0.55 | -2.35~-0.22 | 0.018 |
| T3(G1-G0) | 0.089 | 0.39 | -0.67~0.85 | 0.817 |

**etable 3.3 Between-group and Within-group Pairwise Comparisons Of HADS-A**

|  | **B** | **SE** | **Wald**  **95% CI** | **P** |
| --- | --- | --- | --- | --- |
| Active-tACS Group |  |  |  |  |
| T1-T0 | -2.88 | 0.18 | -3.30~-2.45 | 0.000 |
| T3-T0 | -3.02 | 0.19 | -3.48~3.56 | 0.000 |
| T3-T1 | -0.14 | 0.06 | -2.29~0.00 | 0.058 |
| Sham-tACS Group |  |  |  |  |
| T1-T0 | -1.41 | 0.12 | -1.70~-1.13 | 0.000 |
| T3-T0 | -2.47 | 0.13 | -2.78~-2.16 | 0.000 |
| T3-T1 | -1.06 | 0.06 | -1.21~-0.91 | 0.000 |
| Between-group |  |  |  |  |
| T0(G1-G0) | 0.24 | 0.26 | -0.71~0.85 | 0.356 |
| T1(G1-G0) | -1.22 | 0.19 | -1.59~-0.86 | 0.000 |
| T3(G1-G0) | -0.31 | 0.20 | -0.71~0.09 | 0.131 |

**etable 3.4 Main Effect Test Of HADS-D**

|  | **B** | **SE** | **Wald**  **95% CI** | **P** |
| --- | --- | --- | --- | --- |
| Time |  |  |  |  |
| T1-T0 | -0.99 | 0.09 | -1.16~-0.82 | 0.000 |
| T3-T0 | -1.12 | 0.09 | -1.29~-0.95 | 0.000 |
| T3-T1 | -0.14 | 0.05 | -0.25~-0.02 | 0.014 |
| Group |  |  |  |  |
| G1-G0 | -0.18 | 0.15 | -0.48~0.11 | 0.228 |

**etable 4. Intraoperative and Postoperative Data by the Two Groups^a^**

| **Characteristic** | **Active-tACS**  **(n=84)** | **sham-tACS**  **(n=85)** | **P** |
| --- | --- | --- | --- |
| Intraoperative |  |  |  |
| Duration of surgery, median (IQR), min | 120(90,150) | 120(76.5,150) | 0.568 |
| Duration of anesthesia, median (IQR), min | 137.5(110,173.7) | 142(106,177.5) | 0.971 |
| Estimated blood loss, median (IQR), ml | 50(30,50) | 50(30,50) | 0.518 |
| Infusion quantity, median (IQR), ml | 1300(1100,1600) | 1350(1200,1600) | 0.323 |
| Remifentanil usage, median (IQR), mg | 1.5(1.25,2) | 1.55(1,2) | 0.784 |
| Propofol usage, median (IQR), mg | 300(250,415) | 350(255,440) | 0.181 |
| Blood transfusion, n (%) | 4(4.8) | 4(4.7) | 0.986 |
| postoperative |  |  |  |
| Received oxycodone, n (%) | 12(14.3) | 14(16.5) | 0.694 |
| Time of extubation, median (IQR), min | 20(15,25.75) | 22(20,28) | 0.089 |
| Duration of hospitalization, median (IQR), d | 6(5,7) | 6(6,7) | 0.456 |
| QoR-15 Score on POD3, median (IQR) | 130(127,133) | 129(126.5,131) | 0.114 |

^a^Data presented as median (interquartile range, IQR) were compared using the Mann–Whitney U test. Data reported as the number of patients (%) were compared using the Pearson χ2. Abbreviations: POD3, Postoperative Days 3; QoR-15, Quality of Recovery-15

**etable 5. Mediating Model Examination by Bootstrap**

|  | **Total**  **effect (95% CI)** | **Direct**  **effect (95% CI)** | **Indirect**  **effect (95% CI)** |
| --- | --- | --- | --- |
| Anxiety | 1.143(0.049,2.237) | 0.295(-0.899,1.489) | 0.847(-0.379,1.511) |
| Pain at rest | 1.285(0.203,2.368) | 1.082(-0.130,2.293) | 0.204(-0.393,0.887) |
| Pain at motion | 1.285(0.203,2.368) | 1.129(0.025,2.233) | 0.156(-0.153,0.513) |

Abbreviations: CI, confidence interval.

**etable 6. Incidence of Mild, Moderate and Severe Pain Between the Two Groups^a^**

|  | **Participants, No. (%)** |  |  |  |  |
| --- | --- | --- | --- | --- | --- |
|  | **Active-tACS**  **(n = 84)** | **Sham-tACS**  **(n = 85)** | **OR (95% CI)** | **χ2** | **P** |
| Pain |  |  |  |  |  |
| No pain or Mild pain | 58(69.0) | 35(41.2) | - | 13.26 | 0.000 |
| Moderate and severe pain^b^ | 26(31.0) | 50(58.8) | 0.31(0.17-0.59) |  |  |

^a^Data reported as the number of patients (%) were compared using the Pearson χ2.The total number of people with pain within three days after surgery was counted.

^b^Defined as a numeric rating scale score ≥ 4 points.
